# Non-infectious granulomatous disorders of the upper lip: clinicopathological analysis of 11 patients

**DOI:** 10.1186/s12903-022-02189-z

**Published:** 2022-05-11

**Authors:** Irene Lafuente-Ibáñez de Mendoza, Emmanuelle Vigarios, Béatrice Herbault-Barres, Javier Alberdi-Navarro, Vincent Sibaud, Delphine Maret, José Manuel Aguirre-Urizar

**Affiliations:** 1 Oral Medicine and Pathology Unit, Department of Stomatology II, University of the Basque Country UPV/EHU, Barrio Sarriena s/n, 48940 Leioa, Spain; 2 Department of Oral Medicine, Institut Claudius Regaud, Institut Universitaire du Cancer Toulouse-Oncopole, Toulouse, France; 3 Department of Pathology, Institut Claudius Regaud, Institut Universitaire du Cancer Toulouse-Oncopole, Toulouse, France; 4 Department of Dermatology, Institut Claudius Regaud, Institut Universitaire du Cancer Toulouse-Oncopole, Toulouse, France; 5 Faculté de Chirurgie Dentaire, Université Toulouse III-Paul Sabatier, Toulouse, France

**Keywords:** Granuloma, Upper lip, Systemic, Foreign bodies, Lichen

## Abstract

**Background:**

Non-infectious granulomatous disorders of the upper lip represent a special chapter of oral and maxillofacial pathology. In this work we report a case-series of this process, to analyse its main clinicopathological features and find differential data that allow us improve its diagnosis and understand its pathogenesis.

**Methods:**

We present 11 cases of non-infectious granulomatous disorders of the upper lip, 8 women and 3 men with an age range of 29–84 years, who have been attended at the Oral Medicine Department of the IUCT (France) and the Oral Medicine Unit of the UPV/EHU (Spain). All clinicopathological data were collected in a specific protocol.

**Results:**

We recognized 4 different subtypes of non-infectious granulomatous disorders of the upper lip: (1) associated with Crohn’s disease (1 case), (2) associated with foreign body (2 cases), (3) associated with gingivitis lichenoid-like (4 cases), (4) idiopathic (4 cases).

**Conclusions:**

Clinicopathological differences were identified between these subtypes. A good differential diagnosis is necessary in all cases to rule out the presence of local or systemic etiopathogenic factors.

## Background

“Granuloma” is a specific histopathological form of chronic inflammation, of which two main groups are classically recognised: foreign body granulomas and immune granulomas [1]. Morphologically, the granuloma appears as a round-ovoid formation with a central area with macrophages (epithelioid cells), surrounded by a crown of lymphocytes [2]. Occasionally, these macrophages form multinucleated giant cells, that adopt different patterns [3].

The presence of granulomas in lip biopsies is uncommon, but when observed, it is a key diagnostic finding. Lip granulomatous disorders can correspond to both infectious (tuberculosis, histoplasmosis, etc.) and non-infectious diseases (Crohn's disease, sarcoidosis, foreign bodies, neoplastic processes, etc.) [2, 4, 5].

Since the first description of “orofacial granulomatosis” [6], the diagnosis of non-infectious granulomatous cheilitis of the upper lip has been a challenge in oral and maxillofacial pathology. Several authors [7, 8] have tried to clarify the etiopathogenesis of this particular pathology. Although a causal agent is identified in most of these disorders, there are several subtypes without a recognisable factor [9]. In recent years, many cases of granulomatous cheilitis associated with “lichenoid granulomatous stomatitis” have been described [10, 11].

Commonly the clinical picture of granulomatous cheilitis appears as a non-specific chronic swelling. The granulomatous reaction can be demonstrated with conventional microscopic examination, but special techniques are also important to rule out granulomatous infectious diseases. In all cases, a good clinicopathological correlation is necessary to reach the correct diagnosis and establish the most appropriate therapy [12, 13].

The aim of this study is to present a series of clinical cases diagnosed as non-infectious granulomatous disorder of the upper lip (NIGDUL), in order to analyse its main clinicopathological features and try to find differential data that allow us improve its diagnosis and understand its pathogenesis.

## Methods

In this retrospective observational study, we present a series of cases clinicopathologically diagnosed as non-infectious granulomatous disorder of the upper lip. We used the term “disorder”, as some of them have no, or minimal, inflammatory component.

The patients were studied at the Oral Medicine Service of the Institut Universitaire du Cancer de Toulouse (Toulouse, France) and the Oral Medicine and Pathology Unit of the University of the Basque Country/EHU (Leioa, Spain). These corresponded to 8 women and 3 men, with a mean age of 56.45 years at the time of diagnosis (range: 29–82 years).

A specific clinicopathological protocol was designed for the study, where the main clinical and histopathological data were collected. All patients signed an informed consent form and their personal data were anonymised. The study was approved by the Ethics Committee for Research of the University of the Basque Country and was conducted in accordance with the Helsinki Declaration of 1975, revised in 2000.

In all cases an intraoral incisional biopsy of the swollen upper lip was performed under local infiltrative anaesthesia, and processed in a standard way. All preparations were stained with H&E, and specific techniques (PAS, Zhiel-Neelsen, Grocott) were also performed to rule out the presence of infectious agents. Also, presence of other systemic granulomatous diseases were ruled out.

## Results

We identified 4 different clinicopathological subtypes of NIGDUL: associated with foreign body (2 cases), associated with Crohn’s disease (1 case), associated with gingivitis lichenoid-like (4 cases), and NOS (idiopathic) (4 cases). The main data are shown in Tables 1 and 2.

**Table 1:** Clinical characteristics of the patients with non-infectious granulomatous disorders of the upper lip

NIGDUL subtype	Case	Sex/age	Other oral lesions	Medical history	Treatment
Associated to Crohn’s disease	1	F/29	Ulceration	Crohn’s disease	Anti-TNF (*adalimumab*)
Associated to foreign body	2	F/44	No	Esthetic material (liquid silicone)	Corticotherapy and watchful waiting
	3	F/60		Esthetic material (calcic hydroxyapatite)	
Associated to gingivitis lichenoid-like	4	M/71	Papuloerosive erythema (upper lip, gingiva)	–	Local corticotherapy and watchful waiting
	5	F/28		Allergy to nickel	
	6	F/84		Hepatitis, hypothyroidism, pulmonary fibrosis	
	7	F/77		–	
NOS (idiopathic)	8	F/56	No	–	Corticotherapy and watchful waiting
	9	M/59		–	
	10	M/82		–	
	11	F/32		–	

F: female; M: male; NOS: not otherwise specified

**Table 2:** Histopathological characteristics of the biopsies of non-infectious granulomatous disorders of the upper lip

NIGDUL subtype	Case	Granulomas					Chronic mucositis
		*n*	Localization	Epithelioid cells	Multinucleated giant cells (nucleus, disposition)	Lymphocytic crown	
Associated to Crohn’s disease	1	> 2	Corion, submucosa	Yes	Yes (> 10, disperse)	Intense	Yes
Associated to foreign body	2	> 2	Submucosa	Yes	Yes (2–10, disperse)	Mild	No
	3						
Associated to gingivitis lichenoid-like	4	> 2	Corion, submucosa	Yes	Yes (2–7, disperse)	Intense	Yes
	5						Yes
	6						Yes
	7						Yes
NOS (idiopathic)	8	> 2	Corion, submucosa	Yes	No	Intense	Yes
	9	1	Submucosa			Mild	No
	10	1	Submucosa			Mild	No
	11	1	Submucosa			Mild	No

### NIGDUL associated with foreign body

**Fig. 1:**
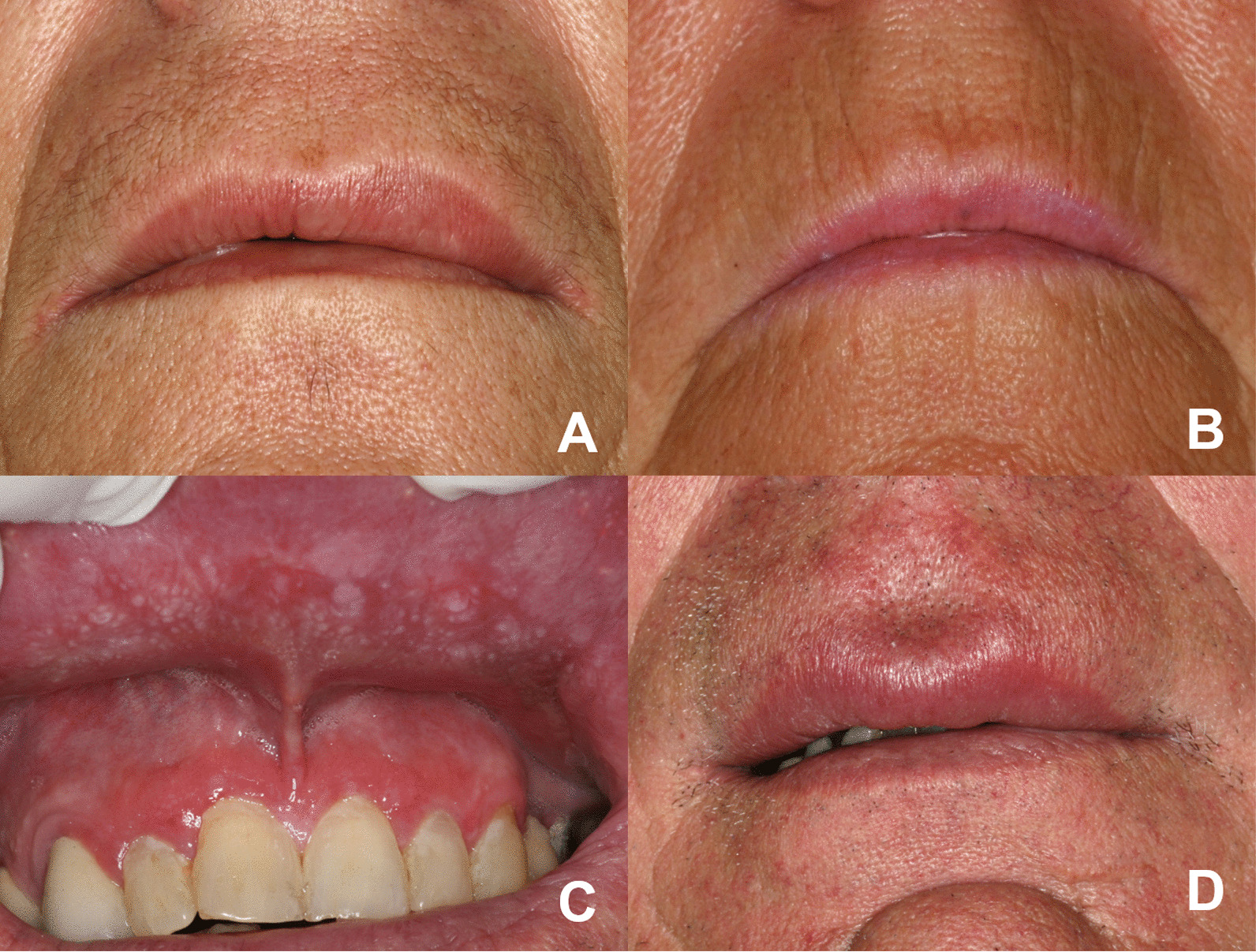
Clinical aspects of the subtypes of NIGDUL: **A** Tumefaction of the left upper lip in the patient with NIGDUL associated with silicone (Case 2), **B** Cheilitis of the left upper lip in the patient with NIGDUL associated with calcic hydroxyapatite (Case 3), **C** Erythematous and white papules in the lip mucosa and symmetric desquamative gingivitis in a patient with NIGDUL associated with gingivitis lichenoid-like (Case 5), **D** Swelling of the upper lip in a patient with NIGDUL NOS (idiopathic) (Case 9)

**Fig. 2:**
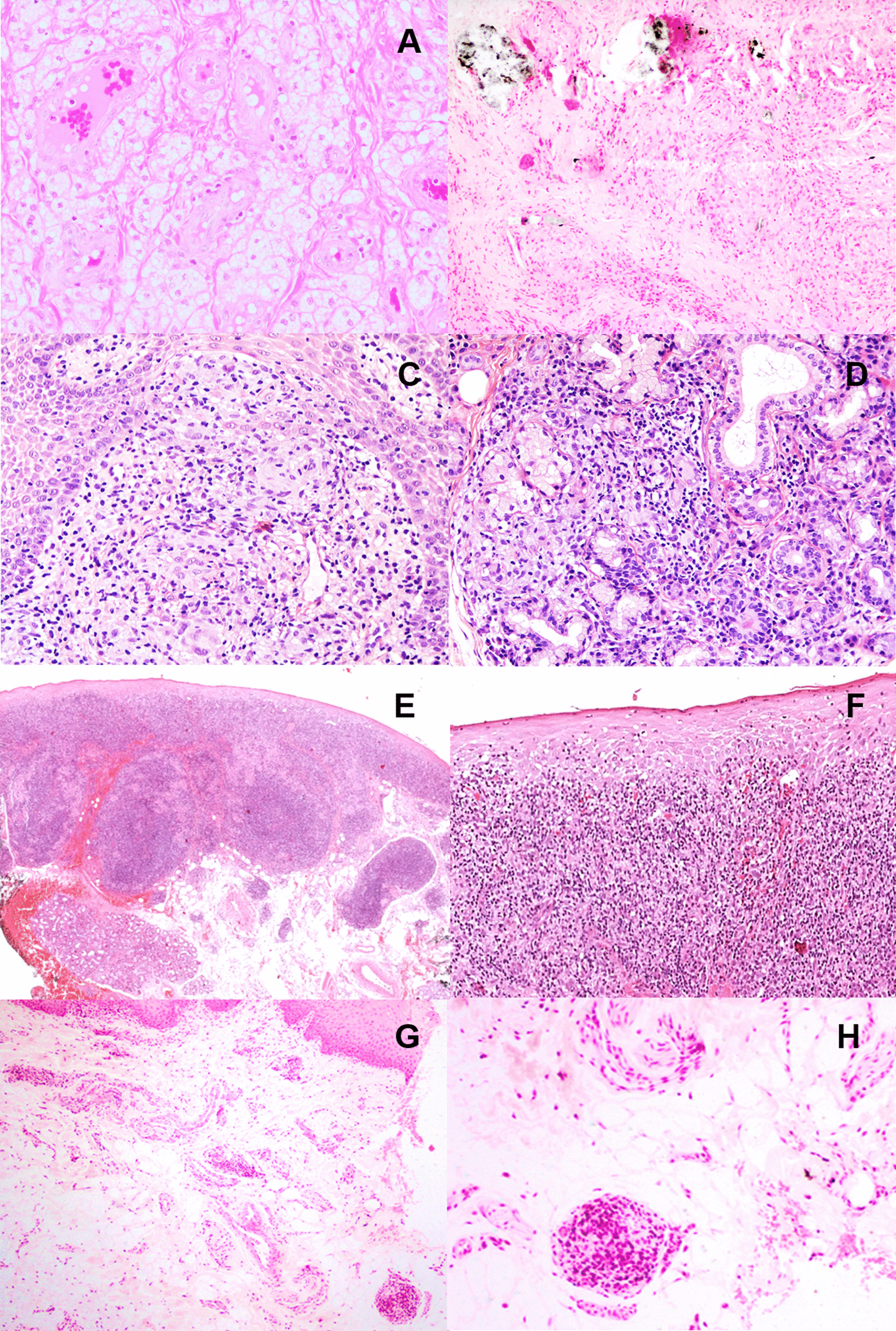
Histopathological features of the subtypes of NIGDUL: **A** “Bubbly” granulomatous reaction in the case of NIGDUL associated with silicone (H&E × 20), **B** Granulomatous reaction and crystalloid foreign body in the patient with NIGDUL associated with calcic hydroxyapatite (H&E × 20), **C** Round granuloma in the lamina propia of NIGDUL associated with Crohn’s disease, showing epithelioid, lymphoid and multinucleated giant cells (H&E × 20), **D** Granulomatous affectation of the salivary gland parenchyma in the same patient (H&E × 20), **E** Multiple granulomas and chronic mucositis (lichenoid-like) in a case of NIGDUL associated with gingivitis lichenoid-like (H&E × 10), **F** Detail of the chronic lymphocytic infiltration in the lamina propia with epithelial atrophy and degeneration of the basal cells (H&E × 20), **G** Small granuloma in the submucosa in a patient with NIGDUL NOS (H&E × 15), **H** Detail of this granuloma with histocytes and lymphocytes (H&E × 40)

These patients were 2 women (44 and 60 years-old) with no past medical history of interest, who had an upper lip swelling of several months of evolution, without erythema (Fig. 1A, B). The clinical differential diagnosis included: orofacial granulomatosis *vs* sarcoidosis and traumatic fibroma. The histopathology showed, in both cases, a non-necrotising granulomatous reaction in the submucosa, with clusters of macrophages, some multinucleated giant cells and no peripheral lymphocytic component. A vacuolated material was recognised within the macrophages of the first case, consistent with liquid silicone (Fig. 2A). On the second, fragments of a greenish-crystalloid foreign material were identified, surrounded by macrophages, compatible with calcium hydroxyapatite (Fig. 2B). Both patients confirmed having been treated for esthetic purposes with these products several years earlier.

### NIGDUL associated with Crohn’s disease

A 29-year-old woman diagnosed with Crohn's disease presented with a swelling of the upper lip with mild mucosal erythema, and an irregular linear ulceration of the buccal mucosa. Incisional biopsies of both lesions were performed. The labial biopsy showed a chronic mucositis and submucositis, in which non-necrotising granulomas were observed (Fig. 2C), which reached the submucosal salivary gland parenchyma (Fig. 2D). These granulomas consisted of rounded clusters of epithelioid cells and multinucleated giant cells, with lymphocytic infiltration. Foreign material was not present. The buccal biopsy also revealed a chronic mucositis with granulomas, similar to the lip lesion.

### NIGDUL associated with gingivitis lichenoid-like

This subtype consisted of 3 women and 1 man, with a mean age of 65 years (range 28–84 years). In all cases, patients reported a long-standing swelling of the upper lip with slight discomfort, accompanied by irregular erythematous and erosive areas with white papules on the labial mucosa, as well as a symmetric desquamative gingivitis lichenoid-like in the upper anterior gingiva (Fig. 1C). One patient reported having a medical history of autoimmune hepatitis treated with immunosuppressants, hypothyroidism on hormone therapy and pulmonary fibrosis. All patients denied previous esthetic treatments and had no other oral lesions. Histopathologically, the biopsies showed an intense chronic lymphocytic mucositis with areas of degeneration of the basal epithelium (Fig. 2E). In the mucosa and submucosa, variable-sized non-necrotising granulomas with epithelioid cells, multinucleated giant cells and lymphocytes, were seen (Fig. 2F). Presence of foreign material was ruled out in all cases, and no dysplastic changes or signs of malignancy were observed. Main systemic granulomatous conditions were discarded in all patients and some laboratory investigations were performed (assessment of angiotensin converting enzyme, levels of folic acid, iron and vitamin B12, etc.).

### NIGDUL NOS (idiopathic)

This subgroup consisted of 2 women and 2 men, with a mean age of 57.25 years (range 59–84 years). All cases presented with a chronic and asymptomatic lip swelling without mucosal or gingival changes (Fig. 1D). None of the patients reported any past medical history of interest, including esthetic treatments. The histopathological study revealed absence, or mild mucositis (Fig. 2G). The non-necrotising granulomas were small and located in the submucosa, acquiring a perivascular disposition. The granulomas showed epithelioid cells, absence of giant cells and variable lymphocytic infiltration (Fig. 2H). Presence of epithelial dysplasia or foreign material was not observed. Presence of foreign material was ruled out in all cases, and no dysplastic changes or signs of malignancy were observed. Main systemic granulomatous conditions were discarded in all patients and some laboratory investigations were performed (assessment of angiotensin converting enzyme, levels of folic acid, iron and vitamin B12, etc.).

## Discussion

It is considered that the initial report of orofacial granulomatosis was made by Melkersson in a patient with facial swelling and paralysis [6]. In 1945, Miescher published the first case of “granulomatous cheilitis” on the upper lip [14]. This granulomatous cheilitis can be accompanied by other oral and facial signs such as ulcerations, gingival overgrowth, lingual lesions, etc., constituting what is known as “orofacial granulomatosis” [4]. This term groups different disorders that characteristically affect the upper lip, and whose clinicopathological differentiation is difficult in many cases [15, 16].

The initial differential diagnosis of granulomatous cheilitis has to be made between infectious and non-infectious processes, but infectious granulomatous cheilitis of the upper lip are rare [17, 18].

Different systemic entities can cause upper lip granulomas and Crohn’s disease is the most emblematic [3]. Crohn’s disease is a chronic inflammatory digestive disorder, whose first clinical manifestation can be oral in up to 30% of cases [19]. The oral presentation is highly variable; however, a cobblestone appearance of the mucosa, lip swelling, linear ulcerations and mucogingivitis are the most common oral findings [20]. In our case, the patient was already aware of her systemic condition. When the oral cavity is the first affected site, final diagnosis is obtained after performing a complete clinical history, intestinal endoscopy and blood tests [19]. Microscopically, the lesions show non-caseating granulomas and chronic inflammation which can extend to involve the salivary gland parenchyma, similar to our case [21]. Interestingly, only the granulomas seen in the case of Chron’s reached the salivary gland parenchyma, which may be helpful for the differential diagnosis of granulomas associated with Chron’s or sarcoidosis with the other subtypes of NIGDUL [3].

The list of esthetic materials associated with granulomatous reaction is extensive: silicone, collagen, hyaluronic acid, calcium hydroxyapatite, etc. [22]. Since patients often do not report the cosmetic procedures they receive, it is very important to perform a good medical history to reach the final diagnosis [22, 23]. Liquid silicone is a hydrophobic polymeric of dimethyl siloxane compounds, whose granulomatous reaction characteristically shows a vacuolar macrophagic appearance [24, 25]. This bubbling or vesicular pattern of the cytoplasm can mimic other pathologies, like liposarcoma and mucoepidermoid carcinoma [26]. Calcium hydroxyapatite (CaHA) is another esthetic filler material consisting of spherical particles embedded in an aqueous gel of carboxymethylcellulose and glycerine [27]. CaHA is an inorganic element of the bone and teeth; yet, it is unknown why more than 10% of patients develop a foreign body reaction [28]. Microscopically, the particles appear surrounded by a histiocytic granulomatous reaction with giant cells [29]. Unlike NIGDUL associated with Crohn’s disease, the granulomatous reaction of this subtype was deeper and did not show chronic lymphocytic inflammation.

The third subtype of our study referred to patients who, in addition to the lip swelling, also presented areas of erythema and erosion on the lip mucosa, with a symmetric desquamative gingivitis lichenoid-like in the upper anterior gingiva. Robinson et al. (2006) [10] were the first to describe this disorder as "lichenoid granulomatous stomatitis". Microscopically, these lesions showed lip granulomatosis, chronic mucositis, areas of basal degeneration and Civatte bodies [10, 11]. In our opinion, “lichenoid granulomatous stomatitis” is not a good name for this disorder, since oral lichenoid disease requires the presence of whitish papular [reticular] lesions in other areas of the oral mucosa, which were not seen in our patients [30]. Furthermore, oral lichenoid disease does not have pathognomonic histopathological features and desquamative gingivitis may appear in other mucocutaneous diseases [31].

Macrophage activation is a complex process involving the complement system (C3b, C5a) and multiple proinflammatory cytokines (TNF, IL-1, IL-6, IL-17 and IFN gamma), via T cell activity [32]. Accordingly, chronic lymphoplasmacytic inflammation maintains the stimulus for the granulomatous response [33]. This granulomatous inflammation encompasses a wide spectrum of histopathological findings, from well-constituted granulomas to clusters of histiocytes admixed with other inflammatory cells [5]. The variation of this granulomatous response depends on different factors, including the location [5], which could explain the different histological aspects of the biopsies of NIGDUL associated with gingivitis lichenoid-like. We believe that, in these cases, there could be contact feedback between the mucosa of the swollen lip and the gingiva. Therefore, it is mandatory to perform a good clinical history to identify local, dental or not, irritative factors.

When faced with a swollen and granulomatous upper lip in which all the previous possible diagnoses have been ruled out, our only option is to call these lip lesions NIGDUL NOS or idiopathic [9]. NIGDUL NOS would be included under the idiopathic orofacial granulomatosis spectrum, which includes cheilitis granulomatosa of Miescher and Melkersson–Rosenthal syndrome (MRS). NIGDUL NOS could be considered as a monosymptomatic form of MRS; thus, it is very important to differentiate both pathologies, since patients with MRS also develop neurologic manifestations, like facial nerve palsy [34]. Several theories have been postulated to explain the development of NIGDUL NOS, such as exposure to food, beverages, toxins, dental materials, etc. [4]. In this condition, there is a patchy, scattered chronic inflammatory infiltrate and the granulomas are most commonly seen in proximity to the vascular structures. We suspect that one of our patients [female, 56 years] could be an early manifestation case of the third subtype [associated with lichenoid-like gingivitis], in which gingival lesions have not yet appeared; thus, precluding the diagnosis of NIGDUL associated with gingivitis lichenoid-like.

## Conclusions

In summary, presence of granulomas in a biopsy of a swollen upper lip requires an exhaustive clinicopathological correlation. When faced with a non-infectious granulomatous disorder of the upper lip, existence of a systemic disease or cosmetic treatment should be initially ruled out. There are some histopathological differences between non-infectious granulomatous disorders of the upper lip that together with clinical features and medical history may indicate the type of NIGDUL. The patients diagnosed with NIGDUL should be monitored periodically to identify the appearance of further intraoral lesions or the development of a systemic process.

## Data Availability

All data generated or analysed during this study are included in this published article [and its supplementary information files].

## Abbreviations

H&E: Hematoxylin and eosin stain.
IFN: Interferon
IL: Interleukin
MRS: Melkersson–Rosenthal syndrome
NIGDUL: Non-infectious granulomatous disorder of the upper lip
NOS: Not otherwise specified
PAS: Periodic acid–Schiff
TNF: Tumor necrosis factor
